# Examination of a paradox: recurrent metastatic breast cancer incidence decline without improved distant disease survival: 1990–2011

**DOI:** 10.1007/s10549-018-05090-y

**Published:** 2018-12-17

**Authors:** Judith Malmgren, Marc Hurlbert, Mary Atwood, Henry G. Kaplan

**Affiliations:** 1 HealthStat Consulting, Inc., Seattle, WA USA; 2 School of Public Health, University of Washington, Seattle, WA USA; 3 Metastatic Breast Cancer Alliance, New York, NY USA; 4 Swedish Cancer Institute, Seattle, WA USA

**Keywords:** Metastatic breast cancer, Breast cancer, Incidence, Risk modeling, Distant relapse-free survival

## Abstract

**Purpose:**

Distant relapse metastatic breast cancer (rMBC) incidence and survival are vital measures of breast cancer diagnosis and treatment progress over time.

**Methods:**

We conducted a retrospective cohort study of stage I–III invasive breast cancer, 1990–2011, follow-up through 2016 [*N* = 8292, rMBC = 964 (12%)] at a community-based institution. Patient and tumor characteristics (treatment, distant recurrence, vital status) from BC registry data were evaluated. Survival analysis and Cox proportional hazards (HzR) with 95% confidence intervals (95% CI) were calculated using distant recurrence and distant disease-specific survival (DDSS) endpoints.

**Results:**

Both 5- and 10-year distant relapse (rMBC) declined over time from 1990–1998 to 2005–2011 [11% to 5%, 16% to 8% (*p* < 0.001)]. Proportionately, HER2 + BC distant relapse decreased 9% and triple negative (HR−/HER2−) increased 8% (*p* = 0.011). In the Cox model, lower stage [stage I: HzR = 0.08 (0.07, 0.10), stage II: 0.29 (0.25, 0.33)], more recent diagnosis years [1999–2004: HzR = 0.60 (0.51, 0.70), 2005–2011: HzR = 0.44 (0.38, 0.52)], HR+ [HzR = 0.62 (0.53, 0.72)], and age 40+ [HzR = 0.81 (0.67, 0.98)] had decreased rMBC risk. Compared to HR+/HER2− BC, triple-negative BC had increased rMBC risk [HzR = 2.02 (1.61, 2.53)] but HER2+ subtypes did not. HR−, age 70+, > 1, or visceral metastases and stage III disease were associated with worse DDSS. DDSS did not improve over time.

**Conclusion:**

rMBC incidence declined over time with decreased HER2-positive distant recurrence, a shift to more triple-negative BC and consistently poor distant disease survival.

## Introduction

An estimated 252,710 new cases of invasive breast cancer will be diagnosed in 2017 with 40,610 breast cancer-related deaths in the same year [1]. Cancer of the breast is the most common type among women and the number two cause of death from cancer among women [2]. Mariotto et al estimate there are 154,794 women living with metastatic breast cancer (MBC) in the US in 2017, 25% with de novo MBC and 75% of with recurrent MBC [3].

In our comparison of de novo (dnMBC) and recurrent distant metastatic breast cancer (rMBC), incidence of rMBC declined over time while dnMBC incidence remained constant [4]. The rMBC/dnMBC ratio changed from 5.5:1 rMBC/dnMBC (85% rMBC) in 1990–1998 to 2:1 in 2005–2010 (67% rMBC). Observational studies have found equivocal results for survival post metastatic breast cancer diagnosis ranging from suggested improvement to no improvement [5–7]. As metastatic breast cancer is incurable and rMBC composes the largest portion of metastatic breast cancer cases, rMBC incidence, type and survival describe the majority of women affected.

Our objective is to identify how distant recurrence (rMBC) has changed over time by patient and tumor characteristics such as initial diagnosis stage, molecular subtype, and diagnosis year, and how distant disease survival has changed over time. An accurate description of today’s recurrent MBC cases and associated survival trends are necessary to evaluate and inform current BC diagnosis and treatment, shape expectations of the future MBC patient population, direct treatment research in the changing MBC landscape, and devise treatment strategies for the new profile of distant recurrence.

## Methods

### Study design

**Fig. 1:**
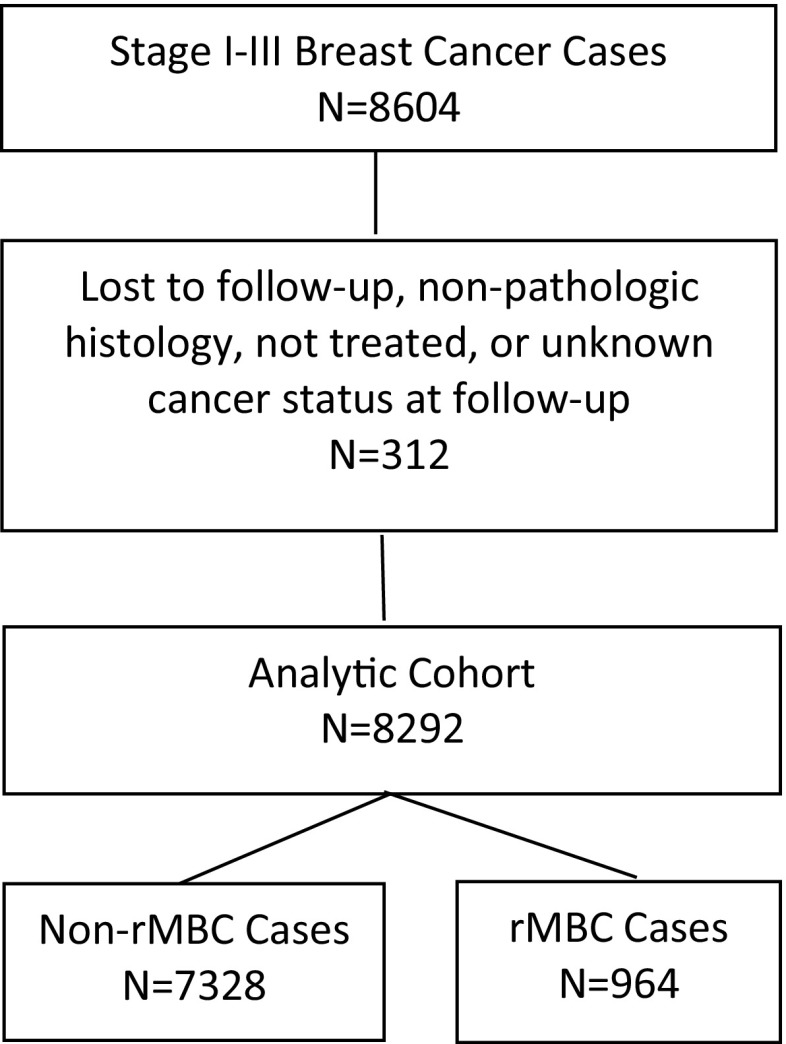
CONSORT diagram

We conducted a retrospective cohort analysis of all first primary AJCC 7 stage I–III invasive BC cases 1990–2011 with follow-up through 2016 for distant recurrent disease (rMBC) [*N* = 8292, rMBC cases = 964] [8]. Patients met National Comprehensive Cancer Network (NCCN) criteria and received treatment for their primary breast cancer [9]. Relapsed MBC (rMBC) was identified after subsequent distant metastatic recurrence post-diagnosis and treatment for primary stage I–III BC. Cases with less than 2 years of follow-up or lost to follow-up (*n* = 25), with non-pathologic histology (*n* = 12), patients not treated for initial breast cancer due to patient choice or precluding factors (*n* = 131), and cases with unknown cancer status at follow-up (*n* = 144) were excluded from the analysis (Fig. 1).

Our institutional breast cancer registry, created in 1990, contains detailed information on diagnosis, pathology, staging, surgery, chemotherapy, radiation therapy, tumor markers, and vital status at follow-up including cause-specific death. Incident BC cases are entered at time of diagnosis into the HIPAA compliant and IRB-approved breast cancer registry. This project was HIPAA compliant and IRB approved by our institution. Patient vital and disease status including date, site and type of recurrence, and date and cause of death is collected prospectively through annual updates by a certified cancer registrar complete through 2016 for this cohort. Follow-up is obtained from (1) electronic chart review, (2) IRB-approved physician directed follow-up letter, (3) institutional cancer registry, and (4) SEER Seattle-Puget Sound Registry [10].

Distant disease recurrence site was restricted to the site(s) at first presentation of distant disease and excluded sites of subsequent disease progression. Dominant distant recurrence sites were (1) soft tissue if distant lymph nodes or skin metastases but not bone or visceral, (2) bone if bone metastases with or without soft tissue but not visceral, and (3) visceral if metastases to organs with or without bone or soft tissue involvement. Either estrogen and/or progesterone receptor positive equaled HR positive and HR negative if negative for both. Breast cancer detection methods from chart notes were (1) patient detection: lump or abnormality discovered by the patient (symptomatic); (2) clinical detection: lump or abnormality discovered during routine physical examination (symptomatic); or (3) mammography detected: lump or breast abnormality from a non-diagnostic mammogram. Self-reported race was coded white/non-white.

Cases were designated rMBC if distant recurrence occurred 3 months or more post initial diagnosis. The time periods (cohorts), 1990–1998, 1999–2004, 2005–2011, were selected by identification of coincident timing of changes in systemic therapy for invasive and metastatic breast cancer including hormone therapy, trastuzumab, taxanes, and neoadjuvant therapy [11].

Time period is used in the models as proxy for treatment change over time based on our NCCN compliant institutions’ use of standard care protocols at time of diagnosis synchronous with guideline changes (Table 1). We evaluated the proportional hazards assumption by plotting ln{−ln(survival)} curves for the ordinal covariate of diagnosis year versus ln (at risk time) and on the basis of Schoenfeld residuals after fitting individual Cox models. We found no evidence suggesting violation of the proportionality assumption graphically or in tests for interaction with the logarithm of survival time.

**Table 1:** Change in systemic therapy 1990–2011: stage I–III (*n* = 8292)

	1990–1998	1999–2004	2005–2011	*p* value
	*N* (%)	*N* (%)	*N* (%)	
Adjuvant chemotherapy (*n* = 4426)	1230 (52%)	1271 (53%)	1925 (55%)	0.134
Taxane therapy (*n* = 2130)	171 (14%)	622 (49%)	1337 (70%)	< 0.001
Neoadjuvant therapy: chemotherapy patients (*n* = 618)	150 (12%)	157 (12%)	311 (16%)	0.001
Hormone therapy: HR+ positive patients (*n* = 5602)	1317 (70%)	1677 (83%)	2608 (87%)	< 0.001
Trastuzumab therapy: HER2 positive patients (*n* = 515)	–	86 (29%)	429 (90%)	< 0.001

Pearson Chi-square (*χ*^2^) testing and mean comparisons (F statistic) were used for initial bivariate and continuous variable comparisons. Distant disease-free interval (DDFI) was time from primary BC diagnosis to distant recurrence (rMBC) and was used for calculating distant relapse-free survival (DRFS). Distant disease-specific survival was from time of distant recurrence (rMBC) to BC-specific death (DDSS). As hormone receptor-positive and negative median time to distant relapse differs, both 5- and 10-year DRFS rates were calculated to accurately capture the differential timing of relapse [12]. Kaplan–Meier estimation was used for 5- and 10-year distant recurrence rates and distant relapse-free survival (log-rank tests). Cumulative rMBC incidence was calculated using one-minus the survival probability from the Kaplan–Meier estimates. Cox proportional hazards models were used to estimate adjusted hazard ratios (HzR) with corresponding 95% confidence intervals (CI) for outcomes distant relapse and distant disease death. All *p* values were two-sided using a 0.05 level of significance. Analyses were conducted using SPSS v.24 [13].

## Results

Out of 8292 invasive breast cancer cases stage I–III at initial diagnosis from 1990 to 2011, 964 cases (11.6%) had distant metastatic recurrence (rMBC). rMBC cases were younger and more often stage II or III than the non-distant recurrence cases (*p* < 0.001) (Table 2). The largest number of rMBC cases occurred in the cohort diagnosed between 1990 and 1998 with fewer distant recurrences in the two subsequent time periods [1990–1998 19%, 1999–2004 11%, 2005–2011 7%, *p* < 0.001]. At initial diagnosis, 74% of rMBC cases were symptomatically detected (*p* < 0.001). rMBC cases were more often high histologic and/or nuclear grade (*p* < 0.001). Average tumor size at initial diagnosis was larger and mean number of positive lymph nodes was greater for rMBC cases (*p* < 0.001). The majority of rMBC cases were hormone receptor positive (74%). Hormone receptor-negative, HER2-positive (HR+/HER2+/HR−/HER2+), and triple-negative (HR−/HER2−) BC cases were more likely to have distant recurrence (Table 2).

**Table 2:** Stage I–III breast cancer w/out distant relapse versus stage I–III with subsequent distant relapse (*n* = 8292)

	Stage I–III	rMBC	*p* value
	(*n* = 7328)	(*n* = 964)	
	*N* (%)	*N* (%)	
Stage			
I	4036 (55%)	163 (17%)	< 0.001
II	2688 (37%)	447 (46%)	
III	604 (8%)	354 (37%)	
Age			
20–39	497 (7%)	140 (15%)	< 0.001
40–49	1680 (23%)	250 (26%)	
50–69	3660 (50%)	428 (44%)	
70–79	1071 (15%)	118 (12%)	
80+	420 (6%)	28 (3%)	
Mean age (range and significance of F statistic)	58 (21–94)	54 (23–93)	< 0.001
Race			
White	6267 (86%)	842 (87%)	0.142
Non-white	1061 (14%)	122 (13%)	
Diagnosis year primary breast cancer			
1990–1998	1917 (26%)	455 (47%)	< 0.001
(row totals)	(81%)	(19%)	
1999–2004	2128 (29%)	260 (27%)	
(row totals)	(89%)	(11%)	
2005–2011	3283 (45%)	249 (26%)	
(row totals)	(93%)	(7%)	
Initial breast tumor detection method			
By patient or physician (symptomatic)	3322 (46%)	706 (74%)	< 0.001
By mammography	3906 (54%)	244 (26%)	
Hormone receptor status at initial diagnosis (*n* = 8169)			
HR+	6182 (86%)	703 (74%)	< 0.001
HER2 status at initial diagnosis (*n* = 5475)*			
HER2+ (HR− or HR+)	690 (14%)	80 (17%)	0.07
HR/HER2 status at initial diagnosis (*n* = 5470)*			
HR+/HER2−	3830 (77%)	287 (60%)	< 0.001
HR+/HER2+	476 (10%)	53 (11%)	
HR−/HER2−	475 (10%)	108 (23%)	
HR−/HER2+	214 (4%)	27 (6%)	
Histologic type primary breast tumor			
Ductal	5924 (81%)	780 (81%)	0.005
Lobular	672 (9%)	113 (12%)	
Lobular/ductal mixed	336 (5%)	37 (4%)	
Other cancer	396 (5%)	34 (4%)	
Nuclear grade initial primary breast tumor			
Low/intermediate	4207 (60%)	357 (41%)	< 0.001
High	2802 (40%)	530 (59%)	
Histologic grade initial primary breast tumor			
Low/intermediate	2492 (36%)	196 (22%)	< 0.001
High	4465 (64%)	703 (78%)	
Tumor size (mean, range, and significance of F statistic)	1.98 cm (0.1–21 cm)	3.67 cm (0.1–20 cm)	< 0.001
Number of positive lymph nodes (mean, range, and significance of F statistic)	0.93 (0–44)	4.44 (0–36)	< 0.001

*Trastuzumab FDA approval 1998, consistent HER2 testing began in 1999

Over time distant relapse decreased among stage I/II BC and shifted to stage III initial invasive BC (*p* = 0.002) (Table 3). More than seventy percent of rMBC cases were hormone receptor positive, consistent over time. Distant relapse among HER2-positive BC declined from 21% in 1999–2004 to 13% in 2005–2011 with a proportional shift to more triple-negative (HR−/HER2−) cases (*p* = 0.011). At metastatic disease diagnosis, the proportion of simultaneous local/regional/distant recurrence, two or more distant metastatic sites, and visceral metastases increased significantly over time (*p* < 0.001).

**Table 3:** rMBC characteristic comparisons by diagnosis year (*n* = 964)

	1990–2011	1990–1998	1999–2004	2005–2011	*p* value
	*N* (%)	*N* (%)	*N* (%)	*N* (%)	
Number of patients	964 (100%)	455 (47%)	260 (27%)	249 (26%)	
Age at initial diagnosis (years)	54 (23–93)	54	55	54	0.217
Stage at initial diagnosis					
I	163 (17%)	87 (19%)	47 (18%)	29 (12%)	0.002
II	447 (46%)	218 (48%)	126 (49%)	103 (41%)	
III	354 (37%)	150 (33%)	87 (34%)	117 (47%)	
Hormone receptor status					
Positive	703 (74%)	335 (75%)	191 (74%)	177 (71%)	0.512
HER2 status* (*n* = 475)					
Positive	80 (16%)		49 (21%)	31 (13%)	0.012
HR/HER2 status* (*n* = 475)					
HR+/HER2−	287 (60%)		138 (60%)	149 (61%)	0.011
HR+/HER2+	53 (11%)		29 (13%)	24 (10%)	
HR−/HER2-	108 (23%)		43 (19%)	65 (27%)	
HR−/HER2+	27 (6%)		20 (9%)	7 (3%)	
First recurrence					
Local/regional	78 (8%)	41 (9%)	17 (7%)	20 (8%)	0.002
Distant	790 (82%)	381 (84%)	221 (85%)	188 (76%)	
Local/regional/distant	96 (10%)	33 (7%)	22 (9%)	41 (17%)	
Number of metastases					
1	573 (59%)	290 (64%)	159 (61%)	124 (50%)	0.001
2+	391 (41%)	165 (36%)	101 (39%)	125 (50%)	
Dominant site of distant metastases					
Bone	328 (35%)	170 (39%)	100 (39%)	58 (24%)	< 0.001
Visceral	522 (56%)	218 (50%)	141 (55%)	163 (67%)	
Soft tissue	87 (9%)	48 (11%)	15 (6%)	24 (10%)	
Distant disease-free interval (mean years)	5.03 (0.17–24.48)	5.64 (0.46–24.48)	5.16 (0.39–16.92)	3.80 (0.17–11.95)	< 0.001

*Trastuzumab FDA approval 1998, consistent HER2 testing began in 1999

**Fig. 2:**
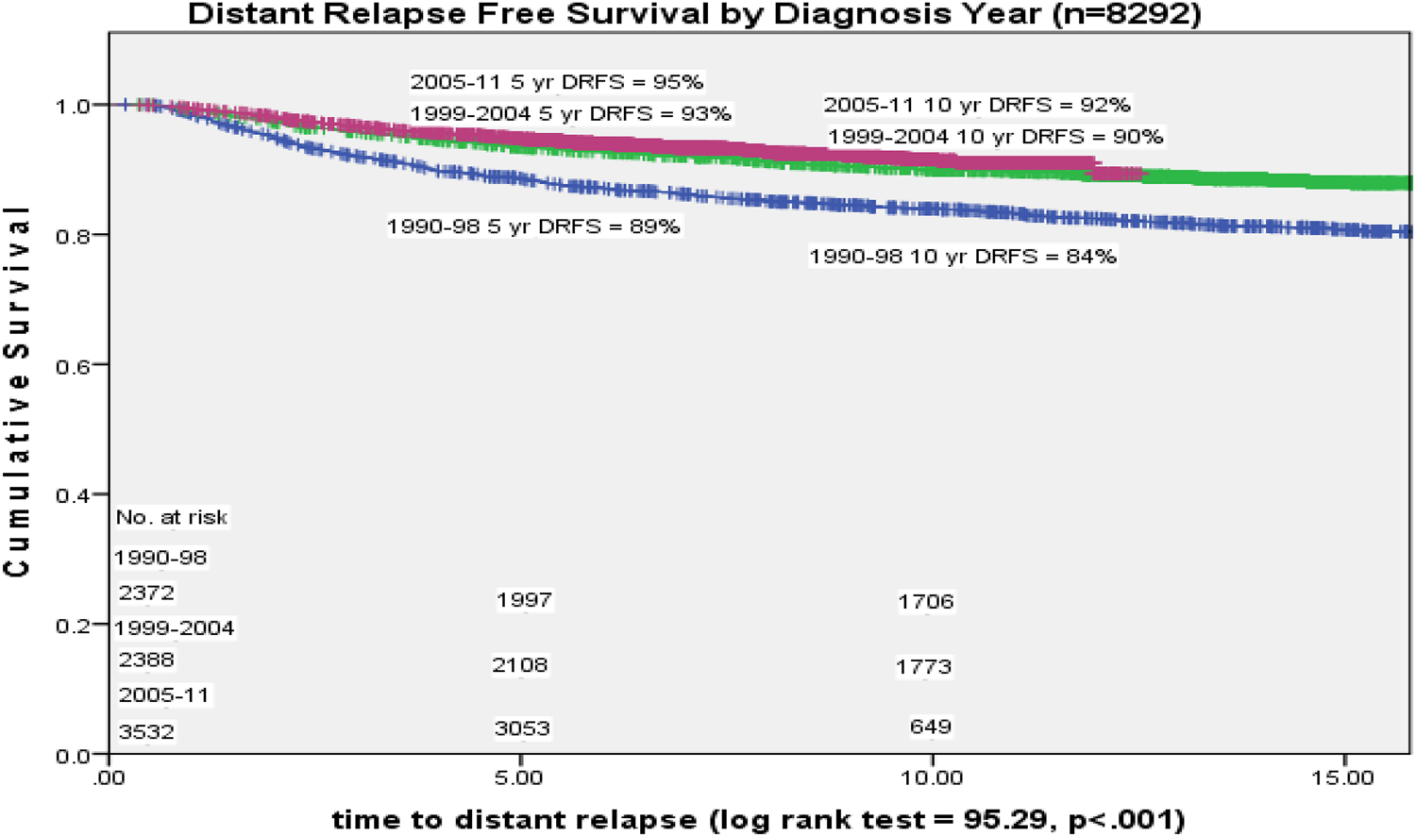
Distant relapse-free survival by initial BC diagnosis year (*n* = 8292)

**Fig. 3:**
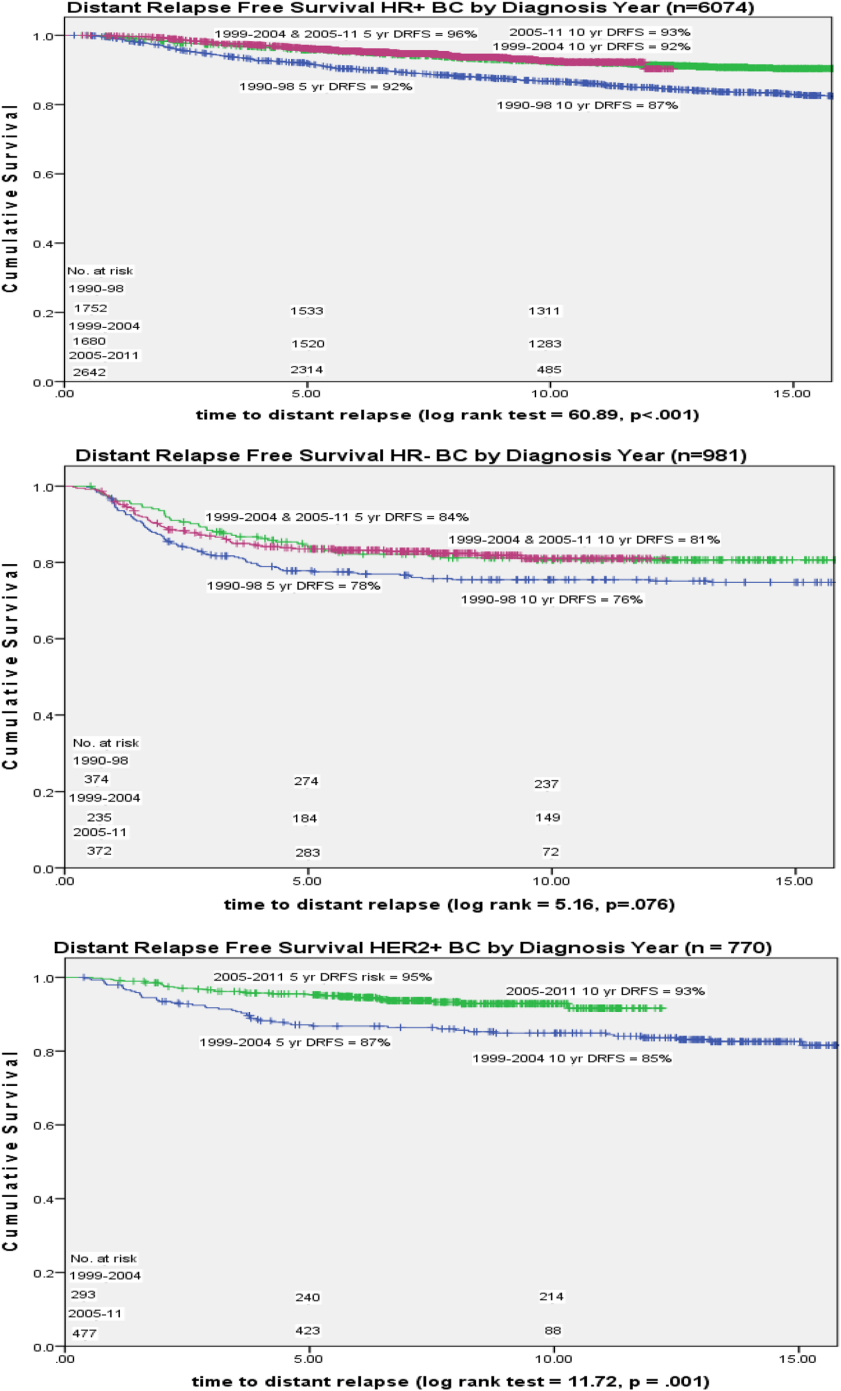
Distant relapse-free survival over time by HR and HER2 status

Median time to distant relapse differed by hormone receptor status [HR+ 4.74 years (range 0.39–24.48 years), HR− 2.83 years (range 0.17–19.25 years) (*p* < 0.001)]. Five and ten-year cumulative rMBC incidence (one-minus-survival) declined over time [5-year 1990–1998 11%, 2005–2011 5%; 10-year 16% to 8% (*p* < 0.001)]. Five- and 10-year DRFS improved over time with 5-year improving  from 89% 1990–1998 to 95% 2005–2011 and 10-year from 84% to 92% (*p* < 0.001) (Fig. 2). Both hormone receptor positive and negative 5-year rMBC cumulative incidence declined from 1990–1998 to 1999–2004 with no change between 1999 and 2004/ 2005–2011 [HR+ 8–4% (*p* < 0.001), HR- 22–16% (*p* < 0.001)] (Fig. 3). Corresponding DRFS improved from 1990–1998 to 1999–2004 for both HR-positive and HR-negative BC [HR+ (92–96%), HR− (78–84%)] with no difference between the latter 2 time periods (Fig. 3). HER2+ 5-year rMBC cumulative incidence declined (13–5%) and 5-year DRFS improved (87% to 95%) from 1999–2004 to 2005–2011 (*p* = 0.001). DRFS differences persisted at 10-years (Fig. 3).

In a cox proportional hazards model adjusted for race; higher risk was associated with higher stage (II and III), diagnosis in the first cohort (1990–1999), HR- status, symptomatic detection, and age 20–39 (Table 4). Conversely lower stage [stage I: HzR = 0.08 (0.07, 0.10), stage II: 0.29 (0.25, 0.33)], more recent diagnosis year [1999–2004: HzR = 0.60 (0.51, 0.70), 2005–2011: HzR = 0.44 (0.38, 0.52)], HR+ [HzR = 0.62 (0.53, 0.72)], and age 40 + [HzR = 0.81 (0.67, 0.98)] were associated with decreased rMBC risk. In a subset analysis, high histologic grade had increased hazard [HzR = 1.27, 95% CI 1.07, 1.50, *p* = 0.007 (*n* = 7674)]. After 1998, compared to HR+/HER2−, TNBC had increased rMBC hazard [HzR = 2.02, 95% CI 1.61, 2.53 (*n* = 5387)] but HER2+ subtypes (HR+/−/HER2+) did not.

**Table 4:** Cox proportional hazards model of distant recurrence among stage I–III BC patients 1990–2011 (*n* = 8292)

	HzR (95% CI)	*p* value
Stage I	Reference	
Stage II	3.18 (2.64, 3.84)	< 0.001
Stage III	10.26 (8.38, 12.56)	
Diagnosis year 1990–1998	Reference	
Diagnosis year 1999–2004	0.61 (0.52, 0.71)	< 0.001
Diagnosis year 2005–2011	0.46 (0.39, 0.55)	
Positive hormone receptor status	Reference	
Negative hormone receptor status	1.54 (1.33, 1.78)	< 0.001
Mammography-detected BC	Reference	
Symptom-detected BC	1.51 (1.29, 1.78)	< 0.001
Age 40 + years	Reference	
Age 20–39 years	1.26 (1.04, 1.51)	0.017
HR/HER2 status comparisons after 1998 (*n* = 5387)		
HR+/HER2−	Reference	
HR+ or HR−/HER2+	0.98 (0.76, 1.26)	0.871
HR−/HER2−	1.97 (1.57, 2.47)	< 0.001

Adjusted for self-reported race and listed by order of entry into the model

*CI* confidence intervals, *HzR* hazard ratio

### Survival

**Fig. 4:**
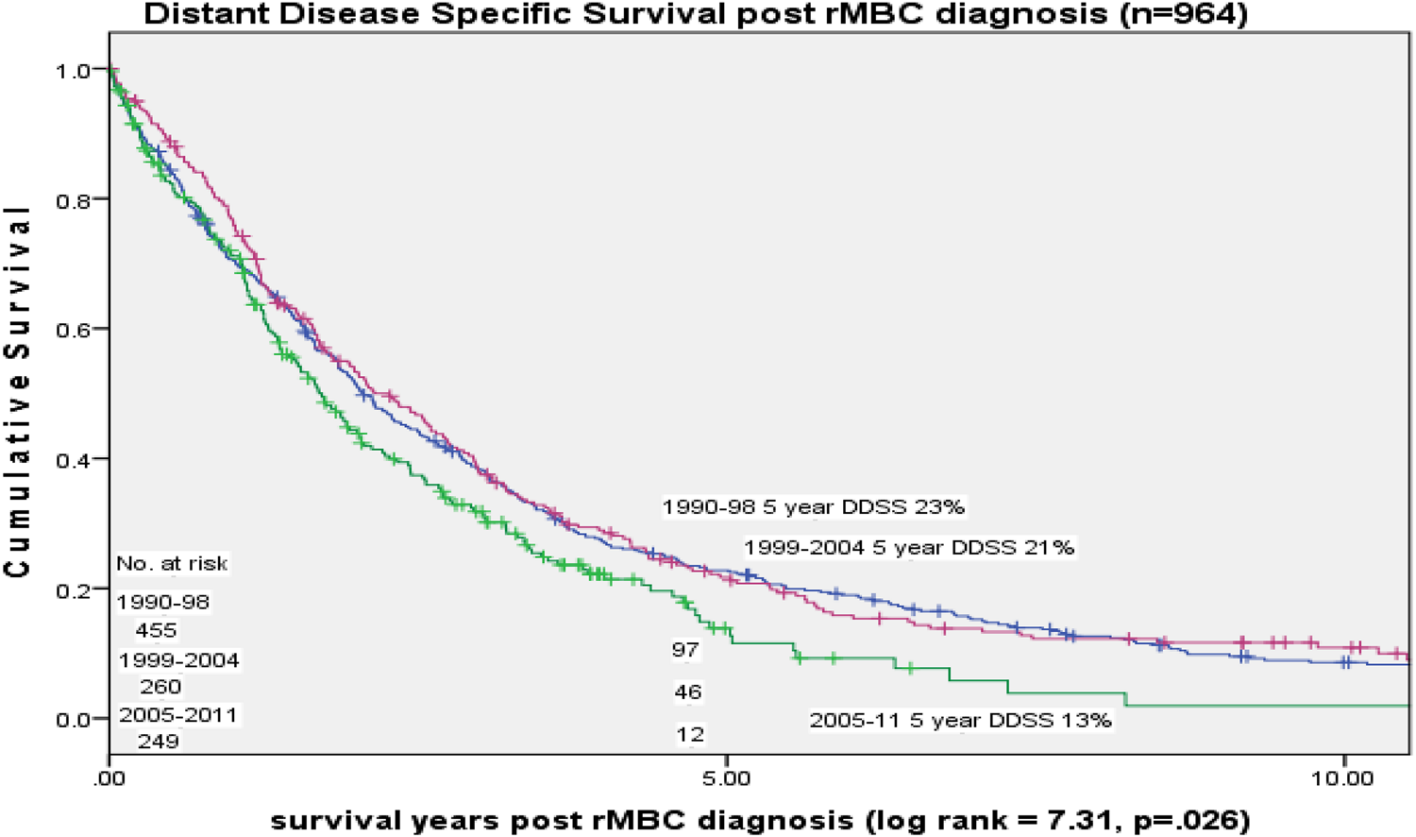
Distant disease-specific survival by initial diagnosis year time period

Five-year distant disease-specific survival declined over time [23% (1990–1998), 21% (1999–2004), 13% (2005–2011) (*p* = 0.026)] (Fig. 4). In a Cox proportional hazards model of DDSS adjusted for race, HR status, older age, number of metastatic sites, type of metastatic site, DDFI, and stage at diagnosis were all significant [HR- HzR = 1.61, 95% CI 1.35, 1.92, age 70 + HzR = 1.94, 95% CI 1.59, 1.94; 2 + metastases HzR = 1.64, 95% CI 1.39, 1.94; bone vs. soft tissue HzR = 1.74, 95% CI 1.30, 2.34; visceral vs. soft tissue HzR = 1.96, 95% CI 1.48, 2.61; DFI < 2 years vs. > 3.7–7 HzR = 1.38, 95% CI 1.01, 1.72; DFI < 2 years vs. 7 + years HzR = 1.91, 95% CI 1.53, 2.39; stage III vs. I HzR = 1.54, 95% CI 1.24, 1.90]. Diagnosis year time period was not significant in the model.

## Discussion

We observed a sharp decline in rMBC incidence over time. Highest risk of distant relapse was associated with earlier time periods (1990–1998, 1999–2004), higher-stage disease at initial diagnosis (Stage II and III), hormone receptor negative or triple negative, younger age (< 40), high histologic grade tumors, or symptomatic disease at time of diagnosis. Disease characteristics at initial diagnosis (higher-stage, negative hormone receptor status, age 70 +) and severity of distant relapse (shorter DDFI, multiple sites, visceral distant disease) were significantly associated with worse distant disease survival. With decreased incidence, rMBC patient characteristics changed by tumor marker subtype (more triple negative, fewer hormone or HER2 positive) and rMBC disease profile (> 1 metastatic site, visceral disease, or combined local/regional/distant at relapse). Distant disease-specific survival did not improve over time.

In a cohort study, Wu et al conducted a multivariate analysis of any recurrence (local, regional, and distant) and observed declining recurrence risk over time associated with cancer detection method, tumor size, number of positive lymph nodes, and triple-negative tumor status [14]. In another cohort study, Cossetti et al observed declining all type recurrence over time, especially among HER2-positive and ER-negative/HER2-negative BC [15]. Chen et al identified stage III, progesterone receptor  negative, and HER2 negative as factors related to distant relapse at a median follow-up of 2.2 years and used their findings to develop a distant recurrence risk score [16]. Using any recurrence, two studies found young age (< 40) associated with higher hazard of recurrence [17, 18]. Other studies found hazard of distant recurrence nearly doubled for tumors found outside of screening (symptomatic disease) [19, 20].

Survival post distant recurrence is rarely examined as few studies have prospective data on outcomes other than singular mortality. In an extensive study of patients treated in adjuvant chemotherapy trials and followed for outcomes (ECOG) (1978–2002), no improvement in survival post distant recurrence was observed over time [7]. Giordano, using institutional cohort data (1974–2000), and Gennari, using clinical trial data (1983–2001), both found suggestive but non-significant survival improvement over time post distant recurrence treatment [5, 6]. In our Cox proportional hazards model, diagnosis year was not associated with post-rMBC diagnosis survival and no survival improvement post-rMBC diagnosis was observed. The link between triple-negative disease and visceral metastasis has been well documented although we found both factors to be independently related to shorter distant disease-specific survival [21].

With the advent of dose-dense adjuvant chemotherapy regimens, taxanes, and effective targeted therapy for both hormone receptor-positive and HER2-positive disease in the adjuvant/neoadjuvant setting, rMBC characteristics have changed coincident with the observed incidence decline [22, 23]. The observed reduction in both HER2 + and HR + rMBC incidence over time indicates increased success of initial targeted therapy with trastuzumab and hormone therapy [24, 25]. Our observation of decreased HER2 + rMBC incidence in the most recent time period is consistent with reported results of improved long-term outcomes after neoadjuvant/adjuvant treatment with HER2-targeted therapy [26]. Improvements in early disease treatment targeted at hormone receptor and HER2-positive disease have reduced overall distant relapse rates but resulted in a new profile of rMBC disease that may be more aggressive with a higher chemoresistant probability [27, 28].

The primary strength of our study is the level of patient and treatment detail with long-term follow-up of all cases for distant recurrence and vital status carried out by a dedicated breast cancer registry staff. A breast cancer registry cohort with continuous follow-up over a 26-year time period provides the opportunity to evaluate changes in incidence and outcomes over a period in which breast cancer treatment has become more effective and personalized. The use of both 5- and 10-year rates of distant disease incidence captures both early (hormone receptor negative) and late (hormone receptor positive) distant disease recurrence, with our DRFS KM plots extending to 15 years of follow-up. The use of hazard modeling allows for simultaneous evaluation of factors presumed to affect the outcomes of interest, distant recurrence and survival time post distant recurrence. Diagnosis year intervals, aligned with timing of known significant treatment changes, were used as factors for therapy advances in the statistical analysis.

Colleoni et al found a lower annualized hazard of recurrence among ER-positive versus ER-negative breast cancer in the first 5 years post-diagnosis which reversed at 10 years post-diagnosis [29]. With less follow-up, our last cohort (2005–2011) findings may be skewed towards more triple-negative and less HR+ distant recurrence due to differential time to distant relapse associated with hormone receptor status. For this reason, we ran additional analysis using only the first two cohorts and Kaplan–Meier plots with at least 10-year follow-up and found no difference in proportional change over time. We do not know if mammography or symptomatic patients participated in regular breast cancer-screening programs. Our observations from an NCCN guideline compliant institution treating a high SES population with less diversity than other metropolitan areas may contribute to better outcomes than those achieved in other parts of the US making our results less generalizable [30, 31].

The observed fifty percent relative decline in distant breast cancer recurrence over time may be related to both improved treatment for initial disease at diagnosis decreasing recurrence risk (hormonal therapy, polychemotherapy, dose-dense chemotherapy, taxanes, and trastuzumab) and stage shift to more early and less late stage disease at diagnosis with improved screening technology and screening program participation [32]. Distant recurrent disease incidence decline over time differed by phenotypic characteristics. We observed a 70% decline in distant disease recurrence among hormone receptor-positive patients and a 50% decline among hormone receptor-negative patients over time. We observed a 60% decline in distant disease recurrence among HER2 positive patients from 1999 to 2006. The differential decline associated with phenotypic subtype creates a new profile of recurrent metastatic breast cancer with fewer HR and HER2-positive cases and relatively more TNBC cases. Ten-year cumulative incidence comparisons to accommodate the longer interval to distant recurrence among HR-positive versus HR-negative disease did not significantly differ from 5-year rates.

The observed change in the current rMBC population to more triple-negative disease, simultaneous local/regional/distant recurrent disease, 2 or more metastatic sites at distant recurrence and visceral disease presents a challenging clinical situation. A more severe metastatic disease profile could be due to better imaging, more TNBC, and distant recurrence among chemoresistant cases. Distant disease survival worsened over time, significant at the .03 level. If high-level guideline compliant care can be universally delivered, on balance rMBC incidence would be expected to decline nationally especially in regards to HR- and HER2-positive appropriate treatment but it is not expected a similar improvement in distant disease survival will be observed.

The development of CDK 4/6 inhibitors for HR+ metastatic disease would be expected to result in longer DDFS for HR+ patients. Their inclusion in neoadjuvant and adjuvant therapy regimens may also reduce the incidence of rMBC [33]. New treatment developments hold promise for extension of life post distant disease diagnosis but the increased challenge of a poor prognosis pool of rMBC cases is a serious problem [34–38]. Clinical challenges include treatment of rMBC patients that are older, previously treated, have multiple metastatic sites and/or visceral metastases at time of distant recurrence. Separate analysis of rMBC and de novo Stage IV MBC in clinical trial evaluation is warranted as these two MBC subtypes may have differential response to treatment [4].

Breast cancer that is higher-stage, symptomatically detected, high histologic grade, triple-negative, or young age at initial diagnosis is at greatest risk for distant relapse. Significant progress has been made in the diagnosis and treatment of breast cancer with guideline compliant treatment as measured by our observation of declining distant recurrence incidence over time. These findings support both screening for early detection and improved treatment over the last three decades. The lack of progress in extending survival for recurrent metastatic breast cancer is disappointing but likely due to the worsening profile of recurrent metastatic disease with tumor chemo-resistance and absence of viable treatment options related to patient or metastatic tumor characteristics. Development of targeted therapies for initial triple-negative disease, screening and breast awareness, treatment guideline compliance, and identification of recurrent metastatic disease treatment are all important goals. At present, guideline adherence and appropriate targeted therapy hold the most potential for distant recurrence reduction and subsequent breast cancer mortality improvement.
